# The effect external and middle ears have in otoacoustic emissions

**DOI:** 10.1016/S1808-8694(15)30826-0

**Published:** 2015-10-18

**Authors:** Christiane Marques do Couto, Renata Mota Mamede Carvallo

**Affiliations:** 1 Adjunct professor of the Speech Therapy course, Faculdade de Ciências Médicas, Universidade Estadual de Campinas - UNICAMP.; 2 Livre-docente certification, adjunct professor of the Speech Therapy course, Faculdade de Medicina da Universidade Estadual de São Paulo- USP.; Universidade Estadual de São Paulo - USP (Sao Paulo State University - USP).

**Keywords:** spontaneous otoacoustic emissions, external ear, middle ear

## Abstract

Characteristics of how external and middle ear resonance frequency can impact the capture of otoacoustic emissions. **Aim:** to study the impact of external and middle ear resonance frequency in otoacoustic emissions. **Study Design:** Prospective, clinical, series study. **Materials and Methods:** Microphone-probe measurements were made in the external ear, together with multifrequency timpanometry distortion product transient otoacoustic emissions in 19 right and 20 left ears from male individuals and 23 right and 23 left ears from female individuals with 17 to 30 years of age. The 85 ears were audiologically normal. **Results:** We did not observe statistically significant associations between the best otoacoustic emission best frequencies and the occluded external and middle ear resonance frequencies. **Conclusion:** Response levels for both transient and distortion product otoacoustic emissions are not influenced by the external and middle ear resonances alone.

## INTRODUCTION

Many factors are responsible for changing the acoustic pattern in the external and middle ear during sound transmission, resulting in a stimulus processed by the central nervous system that is different from the ambient stimulus.^1^

These changes begin at the external ear - with its approximately trumpet shape - and produce resonances that amplify specific frequencies. According to Menezes and Motta,^2^ each structure increases the sound pressure in its natural frequency by about 10 to 12 dB. The first resonant mode in the external ear ranges from 2 500 to 3 000 Hz.^3^

The resonant effects caused by the pinna and ear canal on sound waves that reach the tympanic membrane may be verified by placing a microphone probe close to the tympanic membrane. The sound pressure and impedance values vary when measurements of sound pressure are done with the microphone probe, particularly at high frequencies.^4^, ^5^, ^6^ In normal conditions, impedance of the meatus on the tympanic membrane equals the tympanic acoustic impedance at 3 000 Hz, yielding a condition of maximum auditory sensitivity.^5^, ^7^

The external acoustic meatus guides the waves, linking the external sound field with the tympanic membrane. The tympanic membrane acts as a transducer, transforming sound pressure into mechanical movement, which interacts with the cochlear through the ossicular chain. This chain is formed by three ossicles - the hammer, the anvil and the stirrup - that are linked by flexible connections; transmitted sounds cause the ossicular chain to vibrate. The ensuing mechanical movement is conveyed to the cochlear fluids.

The middle ear is not a perfect transducer; only part of the energy is conveyed, since there is certain opposition to sound. The acoustic impedance in sound transmission from the external acoustic meatus to the cochlea is caused by the interaction between middle ear mass, rigidity and attrition and the impedance of intralabyrinthine fluids. Attrition affects energy transfer uniformly, but mass or rigidity may have a greater or lesser affect depending on the frequency transmitted. There is, however, a middle ear resonant frequency at which the effects of mass and rigidity cancel each other. In normal adults, the mean middle ear resonant frequency is 950 Hz, ranging from 600 to 1 350 Hz.^8^ Multifrequency tympanometry may be used to study this frequency; a tympanometric curve done at the resonant frequency usually presents a characteristic double admittance peak.

The auditory system does not only pick up stimuli passively, but also actively regulates this process, the byproduct of which is sound production. Kemp^9^ has demonstrated this hypothesis by finding sound energy produced within the ear. This energy has been named otoacoustic emission.

Otoacoustic emissions are sounds recorded in the external acoustic meatus that derive from the inner ear activity, specifically the movement of the outer hair cells.^10^ These sounds may be spontaneous or generated by acoustic stimulation. The most frequently used evoked otoacoustic emissions in the clinical setting are the transient stimulus otoacoustic emissions (TOAE), and the distortion product otoacoustic emissions (DPOAE). TOAE are produced by clicks, which are short broad frequency range acoustic transients. DPOAE occur when two pure tones of different frequencies (f1 and f2) are presented simultaneously. The cochlear response is characterized by the occurrence of a third tone whose frequency (2f1-f2) is a distortion product of the stimulus frequencies.

Otoacoustic emissions preferentially reflect the functional status of the cochlea.^11^ They may undergo changes due to external and middle ear structures as ambient sound information reaches the auditory system. Although otoacoustic emissions provide an objective, fast and non-invasive method for assessing pre-neural inner ear function, particularly that of the outer hair cells, they may be absent even in intact cochleae.

Published studies^12^, ^13^, ^14^, ^15^, ^16^, ^17^, ^18^, ^19^, ^20^ have shown that external and middle ear features may interfere doubly on recordings of emissions. Although the sealing condition of buds on the external acoustic meatus and the meatus itself may cause interferences, more emphasis has been given to the role of the middle ear.

According to Elisson and Keefe,^21^ variability in normal middle ear function may explain some of the variations of auditory sensitivity, due to the effect of the middle ear on emissions and the relation of emissions with hearing status. Additionally, diseases of the middle ear generally decrease the amplitude of otoacoustic emissions, and may even obliterate responses.^22^ This is the case of attenuation due to the effects of mass or rigidity in disorders of the ossicular system in the middle ear, such as otitis or otosclerosis.

The simultaneous existence of forward and reverse transmission of stimuli, and the response in certain types of emissions, results in potential interactions between stimuli and responses. Otoacoustic emissions are transmitted from the cochlea to the meatus via the middle ear. Thus, the transmission properties of the middle ear and the external acoustic meatus directly affect the characteristics of emissions. Similarly, the effectiveness of a stimulus used for picking up emissions may also be altered.

According to Margolis and Trine,^23^ although the middle ear conveys sound in both directions, the features of forward and reverse transmission may differ. Forward middle ear transmission establishes the effectiveness of a stimulus reaching the cochlea. The tympanic membrane and the ossicles yield a gain in pressure described as a transformer function for matching impedances. In the reverse direction pressure is lost when energy is transmitted from the cochlea, through the ossicular chain, to the meatus. Mechanical vibrations of the ossicular chain adapt to the moving membrane and yield an airflow pressure wave at the meatus. This sound pressure is inversely proportional to the volume of the meatus. Such influence of the response features may be seen in the opposite direction; the volume of the meatus between the probe and the membrane affects the stimulus intensity and spectrum in forward transmission, since a smaller volume yields higher stimulus sound pressure and a higher resonant frequency at the meatus.

Resonance at the external and middle ear has an important role in transmission, and is easily detected. Wada et al.^13^ concluded that otoacoustic emissions are best detected at the middle ear resonant frequency and in subjects with moderate middle ear mobility.

The purpose of this study was to assess the acoustic features of the external ear (open meatus and meatus occluded by the bud), middle ear and the otoacoustic emissions, given the potential influence of resonances when recording otoacoustic emissions.

Such investigation is extremely important, as the analysis of otoacoustic emissions has been used in detecting early inner ear injuries. Use of otoacoustic emissions may be improved if the evaluator is aware of the role of other structures (external and middle ear) in generating the responses that are gathered.

Traditional immittance testing provides information about the role of the middle ear in this process. However, even though it is possible to correlate absent or decreased otoacoustic emissions response levels with tympanometry, there is no consensus about whether the type of disorder or the middle ear fluid affects emissions;^24^ furthermore, the presence or absence of emissions does not always depend of the type of tympanogram. Tympanometry is not the gold standard for middle ear diagnosis.^25^ A non-invasive assessment of dynamic features (by searching resonant frequencies) may be more discerning when evaluating middle ear function.^26^

Keefe et al.^27^ recommend using a higher information range (0.25 to 8 kHz), in addition to the transference function of acoustic energy by the external acoustic meatus, to assess middle ear status and improve the prediction of hearing loss by otoacoustic emissions.

This study aimed at investigating the influence of external and middle ear resonant frequencies on otoacoustic emission responses.

## MATERIAL AND METHOD

### Ethics

This study abided by the principles of the Helsinki declaration. The Research Ethics Committee of the institution within which the study was conducted assessed and approved the research project (protocol number 536/01).

All subjects consented with the study and the dissemination of its results, according to Resolution 196/96.

### Subjects

The auditory responses of 85 ears were assessed in 20 male subjects (19 right ears and 20 left ears) and 23 female subjects (23 right ears and 23 left ears) aged between 17 and 30 years, with normal hearing in the ears assessed. Subjects had no history of otological disease, metabolic conditions, noise exposure or use of ototoxic drugs, and necessarily had auditory thresholds equal to or better than 25 dBHL, static compliance over 0.2ml, and ipsilateral acoustic reflexes at 500 Hz, 1 000 Hz and 2 000 Hz in the ears that were tested.

### Procedure

The external acoustic meatus was inspected in all subjects to check for any hindrance against the evaluation battery, such as external ear and tympanic membrane conditions. Additional tests consisted of pure tone audiometry from 250 to 8 000 Hz, the speech reception threshold test and the percent index of speech recognition, admittance tympanometry (Ymt) at a probe frequency of 226 Hz, and investigation of ipsilateral acoustic reflexes using stimuli at 500 Hz, 1 000 Hz, and 2 000 Hz. Equipment consisted of a Grason Stadler - GSI-61 two independent channel micro-processed clinical audiometer calibrated according to ANSI 1996 standards, TDH 50 earphones, and a Grason Stadler - GSI 33 version 2 micro-processed middle ear analyzer.

Samples were taken with external ear microphone probe measurements, multifrequency tympanometry and otoacoustic emissions testing.

The microphone probe was used with an open ear approach for measuring sound pressure levels by frequency. The equipment was a Hearing Aid Analyzer -MS 40 (Interacoustics). The MS40 can measure open ear responses in the in situ mode. The insertion depth of the probe tube was 27 mm from the tragus to place the microphone probe at about 8 mm from the tympanic membrane. The patient was placed 0.4 m from the loudspeaker at 0o azimuth. A 70 dBSPL warble tone was used for measurements in the 125 Hz to 8 000 Hz range.

After this measure was taken the point of maximum amplitude on the curve was found; the corresponding frequency was the resonant frequency of the external ear. This frequency was used to calculate the length of the external acoustic meatus (L’) with the equation (1). The same equation was used to calculate the resonant frequency of the occluded meatus. In this case a new length for the canal was used (L”). This length was L” = L’ - 8, caused by inserting the ear bud. Insertion of the bud was 8 mm from the tragus.

In both cases the acoustic meatus was considered as a closed tube (open in one of its ends) with its resonant frequency given by the equation ϖ=ϕ ∑ λ εδνoλλ4= (1), where f = frequency, vS = sound velocity, λ = wavelength, and l = length of the tube (in this case L’ or L”).

The next step was to assess the resonant frequency of the middle ear; multifrequency tympanometry was done in each ear, using the standard program of the middle ear analyzer with three tone frequencies in the immittance probe (226 Hz, 678 Hz and 1 000 Hz). This device can perform tympanometric measures automatically at 50 daPa/s; a printer coupled to this system recorded the results.

The probe frequency varied automatically from 250 to 2 000 Hz at 50 Hz intervals in the initial pressure. Measures of immittance components and phases are stored in the device memory. The first recorded tympanogram was registered in Acoustic Admittance (Ya) mode at a probe frequency of 226 Hz; this was the Test 1. Peak data were noted. A second scan was presented at the Tympanometric Peak (Ya) pressure at 226 Hz. Phase and component measures were again stored. Differences in component values (ΔY, ΔB, and ΔG[1]) and phase values (Δθ[2]) among the first and second frequency scans were calculated and presented on the screen according to frequency variations (from 250 to 2 000 Hz). This was Test 2. The resonant frequency of the ear that was tested was automatically shown by the cursor on the screen. In Test 3 a new tympanogram was traced at the resonant frequency to check the type of curve. At the end of this evaluation admittance measures (Ymt) and the middle ear resonant frequency were recorded.

Subjects also underwent acoustic stimulation with a cochlear emissions analyzer (ILO 92 - version 5.6 - Otodynamics) to observe transient evoked otoacoustic emissions (TOAE) and distortion product otoacoustic emissions (DPOAE). TOAE testing was done using click at the 480 to 5 000 Hz range; the stimulus intensity ranged from 78 to 83 dBpeSPL[3]. The following parameters were noted: reproducibility over 50%, and wave stability over 75%. Responses gathered in four bands centered at 1 000 Hz, 2 000 Hz, 3 000 Hz and 4 000 Hz were used.

DPOAE testing consisted of presenting paired frequencies (f1 and f2)[4] in a ratio such that f2/f1=1.2; the f1 intensity was 65 dBSPL and the f2 intensity was 55 dBSPL. Responses gathered at f2 frequencies of 1 001 Hz, 1 257 Hz, 1 587 Hz, 2 002 Hz, 2 515 Hz, 3 174 Hz, 4 004 Hz, 5 042 Hz and 6 348 Hz were used.

The maximum response frequency was recorded in both emissions tests and named the “best frequency.”

### Statistical analysis

The results were analyzed statistically by comparing the variables as follows:

resonant frequency of the occluded external ear - “Occluded External Ear Resonance”;

resonant frequency of the middle ear - “Middle Ear Resonance”;

frequency with the highest TOAE response level - “Best TOAE Frequency”;

frequency (f2) with the highest DPOAE response level - “Best f2 DPOAE Frequency”.

The linear regression for correlating the variables and the Analysis of Variance (ANOVA) were done. The significance level was 5%.

## RESULTS

Table 1 shows the minimum, mean, maximum and median values of the middle ear resonant frequency, the occluded external ear resonant frequency, and the frequency at which TOAE and DPOAE values were highest or detected most clearly (best frequency) in 85 ears.

**Table 1:** Minimum (MIN), maximum (MAX), mean (MEAN), and median (MEDIAN) values, and the standard deviation (SD) of variables.

	MIN (Hz)	MEAN (Hz)	MEDIAN (Hz)	SD (Hz)	MAX (Hz)
Middle Ear Resonant	550,00	972,35	972,35	184,35	1450
Occluded External Ear Resonant	2212,58	3641,17	3641,17	978,20	8549.47
Best TOAE Frequency	1000,00	1811,76	1811,76	1005,86	4000,00
Best f2 DPOAE Frequency	1001,00	4682,36	4682,36	1627,97	6348,00

First of all, the mean and median values for the best TOAE frequency were 1 811 Hz and 1 000 Hz; for the best DPOAE were 4 862 Hz and 5 042 Hz; for the occluded external ear resonant frequency were 3 641.17 Hz and 3 552.17 Hz; and for the middle ear resonant frequency were 972.35 Hz and 950 Hz.

A relation may be seen between the TOAE/DPOAE and the resonant frequencies; there is a concentration of higher TOAE levels and middle ear resonants at medium frequencies, and of higher DPOAE levels and external ear resonants at higher frequencies.

**Chart 1:**
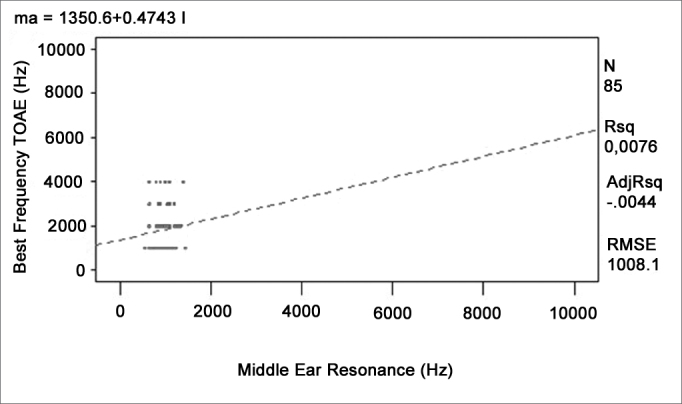
Relation between the best TOAE frequency and the middle ear resonant frequency.

**Chart 2:**
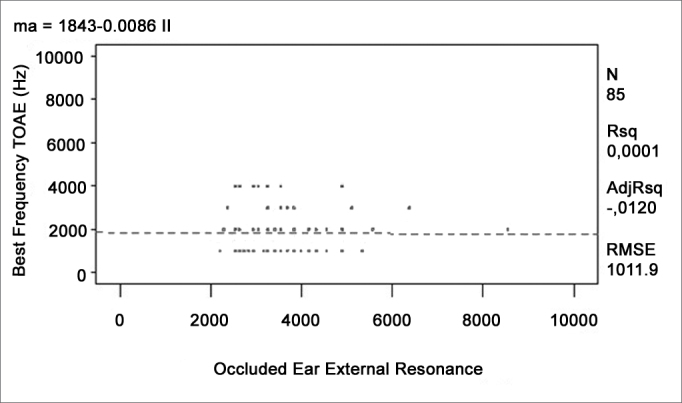
Relation between the best TOAE frequency and the occluded external ear resonant frequency.

**Chart 3:**
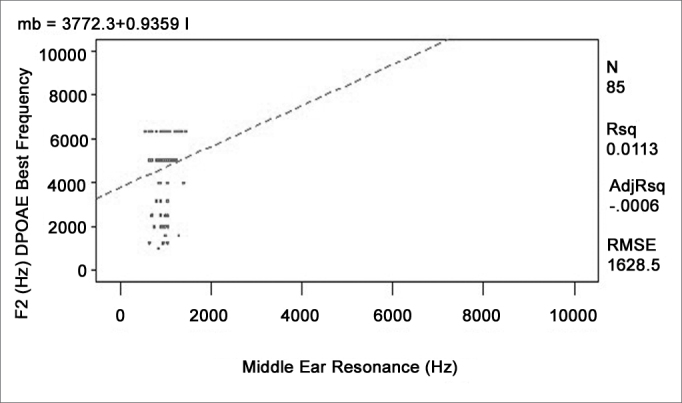
Relation between the best f2 DPOAE frequency and the middle ear resonant frequency.

**Chart 4:**
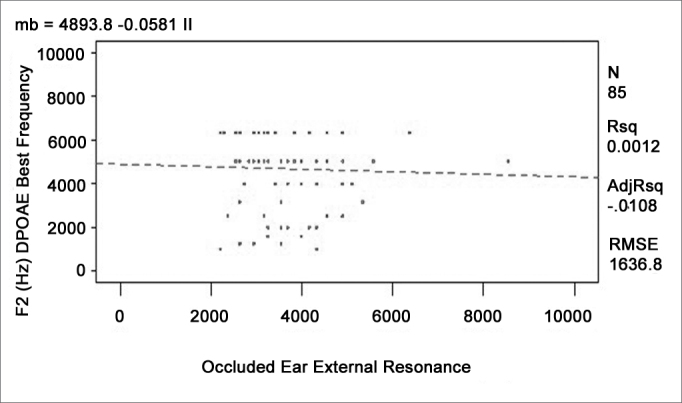
Relation between the best f2 DPOAE frequency and the occluded external ear resonant frequency.

A regression analysis to adjust four different linear models was done to verify whether any given external and middle ear resonant frequency could affect the higher response levels found at certain frequencies in otoacoustic emissions recordings. The ANOVA was carried out to verify the significance of the relation among variables. Table 2 shows the R-square values and the p-values. Chart 1, Chart 2, Chart 3, Chart 4 show the relation among variables.

**Table 2:** Value of the mean quadratic residue, the f value, and the confidence interval for the function of each variable pair

	Variables	R-square	p-model	p-intercept	p-variable
TOAE Hz)	Middle Ear Resonance (Hz)	0,0076	0,4283	0,0245	0,4283
TOAE (Hz)	Occluded External Ear Resonance (Hz)	0,0001	0,9397	<0,0001	0,9397
DPOAE (Hz)	Middle Ear Resonance (Hz)	0,0113	0,3337	0,0002	0,3337
DPOAE (Hz)	Occluded External Ear Resonance (Hz)	0,0012	0,7513	<0,0001	0,7513

None of the models presented satisfactory adjustment levels to confirm the hypothesis that emissions could be affected directly by external or middle ear resonance features. All models revealed that R-square values were below 5% and the p-values were over 30%, showing lack of adjustment.

## DISCUSSION

In our study the highest concentration of the best TOAE frequencies was at 1 000 Hz. This concentration coincides with the highest middle ear resonant frequency concentration, from 900 to 1 050 Hz. Kemp et al.^12^ have suggested that the spectrum of transient emissions frequencies reflect the middle ear transference function, with best transmission efficiency in the 1 000 to 1 500 Hz range and transmission loss of about 12 dB per octave for low and high frequencies.

According to Kemp,^28^ TOAE responses are stronger and more easily detected in the 1 to 4 kHz range; a normal adult ear yields weak TOAE (less than 3 dBSPL) and no substantial responses above 4 kHz. Mor and Azevedo^29^ also found a uniformly occurring TOAE response between 1 000 and 4 000 Hz, decreasing at 5 000 Hz.

Our data shows that the highest concentration of the best DPOAE frequencies was at 5 042 Hz. According to Keefe,^19^ the minimum audible mean pressure (MAP) at the tympanic membrane increases slightly at frequencies over 5 kHz, while distortion product thresholds increase abruptly from 4 to 8 kHz. It is thought that both these response levels are affected by a common factor. Middle ear cavity resonance may be involved, and may be a necessary component in producing forward and reverse transmission. However, the equipment we used measure the middle ear resonant frequency only in the 250 to 2 000 Hz range, which limited any correlation studies at higher frequencies.

Although many authors have reported interferences of middle ear features on otoacoustic emissions, confirming that even though otoacoustic emissions are generated within the cochlea they may be decreased when the sound conduction system is compromised, most of the experiments have found only interferences caused by pressure variations of the tympanic membrane^15^, ^30^ or middle ear structural disorders.^16^, ^18^ Traditional immittance measures done when disorders were present (such as otitis, otosclerosis, or disarticulated ossicles) are positively correlated with changes in emissions. However, often middle ear disorders cause a disproportional impact on the expression of otoacoustic emissions. The presence or absence of emissions does not always depend on the type of tympanogram. Evidence of abnormal middle ear function gathered by conventional tympanometry appears to be a poor predictor of emission status. Investigating the dynamic features of the middle ear (resonant frequency) may help discern diseases in patients with middle ear disorders, thus affecting the emissions status.^26^ This may be attained with equipment such as that developed by Wada et al.;^13^, ^14^ these authors have developed a new system to measure the dynamic features of the middle ear under physiological conditions by using an impedance meter with frequency scanning.

Still according to Wada et al.,^13^, ^14^ the paucity of research on the variability of normal conditions is mainly due to a lack of equipment that might provide further information about the middle ear status compared to the conventional middle ear analyzer. More sophisticated equipment make it possible to assess the influence of the middle ear resonant frequency on otoacoustic emission recordings. Studies^13^, ^14^ done with this type of equipment have shown that the sound energy coming to the external acoustic meatus is transmitted more efficiently to the cochlea at the middle ear resonant frequency.

Wada et al.^13^ have also found that otoacoustic emissions evoked by tone bursts are best detected (highest response level and best otoacoustic emissions frequency) at the middle ear resonant frequency (0.8 to 1.5 kHz). At this frequency the membrane vibrates with maximum amplitude, and sound energy coming from the external acoustic meatus is efficiently transmitted into the cochlea. These authors have stated that this relation may have been influenced by the frequencies that were investigated, since in this case emissions were measured only up to 2 kHz.

Wada et al.^14^ assessed the interference caused by the middle ear resonant frequency on click-evoked and distortion product otoacoustic emissions. They found a relation between the best click-evoked or distortion product otoacoustic emission frequency with the middle ear resonant frequency. This correlation was strongest in click-evoked emissions when the stimulus was linear; a non-linear stimulus may eliminate the middle ear effect in click-stimulated responses. The response level of distortion product otoacoustic emissions was higher when the f2 frequency was around 1.2 kHz, a value that is similar to the middle ear resonant frequency. The same occurred when the mean geometric frequency (f1f2)1/2 equaled the middle ear resonant frequency.

There are, however, certain differences between our study and that of these authors. Analysis of the middle ear resonant frequency was done with different devices; although both used a scanning frequency between 0.1 and 2 kHz as the stimulus, the equipment used by those authors expressed its results in dBSPL, rather than in compliance or impedance units. Similarly, those authors recorded more emissions with smaller intervals between frequencies. Additionally, tone burst evoked emissions were recorded only between 0.5 and 2 kHz in the first paper. In the second paper, TOAE were investigated using linear and non-linear stimuli, which differed from the study that used only non-linear stimuli. A further difference was that a different ILO system was used for investigating distortion product otoacoustic emissions.

No coincidence with best emission frequencies were found in the resonant frequency of the occluded external ear; its concentration was in the 3 000 to 4 000 Hz range. There are no reports in the literature about any concentration of highest response level frequencies of emissions and the resonant frequency of the external ear, although it has been pointed out that lower meatus volumes could have higher resonant frequencies, resulting in stronger stimuli.^23^

Coube^31^ has reported that negative DPOAE values (-3.6 dBSPL) at 3 kHz could be considered as normal hearing; only positive values were observed at 1, 2, 4 and 6 kHz. This author believes that further research is needed to interpret these findings. The authors of the present paper believe that the influence of external ear resonant frequency may be involved in this finding.

There were no statistically significant relations between the best otoacoustic emissions frequency and the occluded external ear resonant frequency. There was, however, a concentration of higher TOAE levels at middle frequencies and of higher DPOAE levels at high frequencies; similarly with middle ear resonants at middle frequencies and occluded external ear resonants at high frequencies. In the case of the best TOAE frequency, the concentration was similar to the middle ear resonant (1 000 Hz).

Certain conditions of this study may have limited verification of the relation among variables. The main difficulty is the resolution of measurements. There is no possibility for assessing intermediate values when analyzing emissions responses by ranges, even though the variables were continuous. Furthermore, stationary wave artifacts result in loss of precision when specifying the non-linear pressure.

The data suggest that the response levels of otoacoustic emissions are not primordially affected by the primary resonance. It should be said that there are many resonance modes, as well as anti-resonance, which may coincide with the depressions in the response level of otoacoustic emissions. Additionally, resonances are only another element among the complex factors involved in simultaneous forward and reverse transmission of stimuli and responses. External and middle ear differences alter the transference function of the middle ear and affect physiological measures, such as otoacoustic emissions.

According to Keefe and Levi,^32^ the admittance level is affected by the area and length of the meatus, by resonances on the wall of the meatus, by the inter-function of controlled compliance and inertial effects in the middle ear, and by the presence of losses. The pressure response varies according to the acoustic features in a system into which a probe is inserted. Ear features affect the transference of force from a free field to the middle ear.

Admittance from the source to emissions is the middle ear entry admittance. The pressure response measured by the probe is described by the transmission function from the acoustic meatus and the tympanic membrane to the probe; this transformer function depends on the cross-sectional are and the length of the meatus and the source impedance of the probe. According to Pruria,^33^ measures of forward and reverse pressure gains in the middle ear indicate that the emissions-generating mechanism (clicks or distortion product) is frequency-dependent.

At the same time, knowledge about otoacoustic emissions is still recent. After being generated in the cochlea, otoacoustic emissions undergo interferences along the path to the probe, which may indicate minor impedance changes in the conduction system. They thus reflect the status of the auditory system, whose function it is to pick up, conduct and amplify sound vibrations, and to constantly maintain the best transducing conditions for high yield rates. The result is to transfer the information contained in sound energy to specialized sensory cells, which convert vibrations into electrical impulses for transmission to the auditory cortex.

Such a rich and effective system necessarily contains many partially unknown functions. This study, along with others that deal experimentally with these and other elements, may probably contribute to provide important information about the auditory system integration.

## CONCLUSION

There was a concentration of higher TOAE response levels at middle frequencies, and of DPOAE at higher frequencies. There was also a concentration of middle ear resonant frequencies at middle frequencies, and of occluded external ear resonant frequencies at high frequencies. In the case of TOAE, there was a coincident concentration between the best response frequency and the middle ear resonant frequency close to 1 000 Hz.

In spite of these results, out study was unable to demonstrate any significant influence of certain occluded external ear and middle ear resonant frequencies on the response level at specific TOAE and DPOAE frequencies.
